# Coral Disease and Ingestion: Investigating the Role of Heterotrophy in the Transmission of Pathogenic *Vibrio* spp. using a Sea Anemone (*Exaiptasia pallida*) Model System

**DOI:** 10.1128/aem.00187-23

**Published:** 2023-05-16

**Authors:** William A. Norfolk, Carolina Melendez-Declet, Erin K. Lipp

**Affiliations:** a Department of Environmental Health Science, University of Georgia, Athens, Georgia, USA; University of Delaware

**Keywords:** coral disease, transmission, ingestion, *Vibrio*, colonization, anemone

## Abstract

Understanding disease transmission in corals can be complicated given the intricacy of the holobiont and difficulties associated with *ex situ* coral cultivation. As a result, most of the established transmission pathways for coral disease are associated with perturbance (i.e., damage) rather than evasion of immune defenses. Here, we investigate ingestion as a potential pathway for the transmission of coral pathogens that evades the mucus membrane. Using sea anemones (*Exaiptasia pallida*) and brine shrimp (*Artemia* sp.) to model coral feeding, we tracked the acquisition of the putative pathogens, Vibrio alginolyticus, V. harveyi, and *V. mediterranei* using GFP-tagged strains. *Vibrio* sp. were provided to anemones using 3 experimental exposures (i) direct water exposure alone, (ii) water exposure in the presence of a food source (non-spiked *Artemia*), and (iii) through a “spiked” food source (*Vibrio*-colonized *Artemia*) created by exposing *Artemia* cultures to GFP-*Vibrio* via the ambient water overnight. Following a 3 h feeding/exposure duration, the level of acquired GFP*-Vibrio* was quantified from anemone tissue homogenate. Ingestion of spiked *Artemia* resulted in a significantly greater burden of GFP-*Vibrio* equating to an 830-fold, 3,108-fold, and 435-fold increase in CFU mL^−1^ when compared to water exposed trials and a 207-fold, 62-fold, and 27-fold increase in CFU mL^−1^ compared to water exposed with food trials for V. alginolyticus, V. harveyi, and *V. mediterranei*, respectively. These data suggest that ingestion can facilitate delivery of an elevated dose of pathogenic bacteria in cnidarians and may describe an important portal of entry for pathogens in the absence of perturbing conditions.

**IMPORTANCE** The front line of pathogen defense in corals is the mucus membrane. This membrane coats the surface body wall creating a semi-impermeable layer that inhibits pathogen entry from the ambient water both physically and biologically through mutualistic antagonism from resident mucus microbes. To date, much of the coral disease transmission research has been focused on mechanisms associated with perturbance of this membrane such as direct contact, vector lesions (predation/biting), and waterborne exposure through preexisting lesions. The present research describes a potential transmission pathway that evades the defenses provided by this membrane allowing unencumbered entry of bacteria as in association with food. This pathway may explain an important portal of entry for emergence of idiopathic infections in otherwise healthy corals and can be used to improve management practices for coral conservation.

## INTRODUCTION

In recent years, coral reefs have experienced unprecedented decline with regular mass mortality events occurring annually across the globe (1). As ecosystem engineers, hermatypic corals produce the foundation of reef habitats by creating the critical three-dimensional structure that defines the reefscape (2). The loss of key coral species causes a decline in habitat complexity leading to a subsequent loss of biodiversity and reef ecosystem services (e.g., coastal protection, fisheries stability, and ecotourism) (1, 3–5). While coral decline can be attributed to many factors, including global climate change, pollution, eutrophication, anthropogenic development, and overfishing, coral disease remains one of the most prominent causes of regional mortality events worldwide (6–10).

Understanding disease transmission, or how a pathogen spreads between individuals in a susceptible population, is a critical component for the management of infectious disease. A mechanistic understanding of the processes related to pathogen movement from reservoirs, through the environment, and into a susceptible host can provide insight for the prediction of disease outbreaks. Prior investigations of coral disease transmission have demonstrated the importance of direct contact, vector transmission, and waterborne transmission via preexisting lesions (reviewed by Shore & Cadwell, 2019 [11]). However, few studies have directly investigated the mechanisms of waterborne transmission, or ambient transmission via exposure in the water column, in uninjured healthy corals. Direct acquisition of pathogenic bacteria from the water column is impeded by the mucus membrane, which creates a semi-impermeable physical and biological barrier surrounding the coral tissue and by ciliary flows that create microscale water currents reducing the efficacy of pathogen chemotaxis (12–14). Thus, in the absence of injury where these systems are degraded, pathogens must overcome these defenses or utilize alternate portals of entry to establish infection.

Two recent studies have suggested that direct bacterial ingestion or ingestion of zooplankton may play an important role in the transmission of coral disease. Certner et al. (2017) (15) demonstrated that white-band disease (WBD) transmission can be facilitated through zooplankton ingestion following incubation in tissue homogenate from diseased corals. In a similar vein, Gavish et al. (2021) (16) utilized a microscale visualization system to observe colonization of *Pocillopora damicornis* by Vibrio coralliilyticus from ambient seawater, suggesting that ingestion may be a primary route of entry for the pathogen. Corals support their carbon and nutrient needs through the mutualistic relationship with their algal symbionts and through direct feeding. Heterotrophy provides up to 35% of a healthy coral’s daily metabolic needs and up to 100% in bleached corals, largely by nighttime feeding on zooplankton (17, 18). While Gavish et al. (2021) (16) demonstrates the viability of pathogen acquisition via direct ingestion of bacteria, preferential grazing of zooplankton, which are known to be colonized by bacteria (and *Vibrio* in particular [19]), may represent an important exploitable pathway for pathogenic microbes to gain entry to a coral host. We hypothesize that pathogen-colonized zooplankton may serve as a foodborne vector for disease transmission in uninjured corals.

*Vibrio* spp. are ubiquitous aquatic bacteria frequently identified as the causative or putative agents of coral disease (Table 1) (20). As indigenous microorganisms, or bacteria that exist naturally as a part of the ambient microbial community, *Vibrio* exhibit complex interspecies interactions that allow them to inhabit a broad range of ecological niches in the environment (21). Of particular note is the association between *Vibrio* spp. and chitinous zooplankton (19, 21). Prior studies of *Vibrio* populations frequently associate total *Vibrio* and/or specific *Vibrio* spp. with plankton presence (22–28). This association has been suggested to facilitate bacterial dispersal (19, 29), reduce bacterivore predation (30, 31), and/or enable the utilization of chitin as a substrate (19, 32, 33).

**TABLE 1:** Published occurrences of *Vibrio* spp. as causative or associated agents of coral disease^*a*^

*Vibrio* spp.	Disease type	Disease name or Description	Affected host	Citation(s)
Vibrio coralliilyticus	White disease^*b*^	Bacterial bleaching disease	*Pocillopora damicornis* and Oculina patagonica	71, 72
		*Montipora* white syndrome^*c*^	*Montipora capitata*	73, 74
		Indo-Pacific white syndrome^*c*^	*Acropora cytherea,* Montipora aequituberculata, and *Pachyseris speciosa.*	75
Vibrio mediterranei (Vibrio shiloi)	White disease^*b*^	Bacterial bleaching disease	Oculina patagonica	76, 77
Vibrio harveyi (*Vibrio charchariae*)	White disease^*b*^	White band disease	*Acropora cervicornis*	78, 79
		White syndrome^*c*^	*Pocillopora damicornis* and *Acropora* spp.	80
	Yellow disease	Yellow band disease^*d*^	*Orbicella faveolata*	81
Vibrio alginolyticus	White disease^*a*^	*Porites andrews*i white syndrome	*Porites andrewsi*	82
	Yellow disease	Yellow band disease^*d*^	*Orbicella faveolata*	81
Vibrio natriegens	White disease^*a*^	*Porites* ulcerative white spot disease	*Porites cylindrica*	83
Vibrio owensii	White disease^*a*^	*Montipora* White Syndrome^*c*^	*Montipora capitata*	74, 84
Vibrio parahaemolyticus	White disease^*a*^	*Porites* ulcerative white spot disease	*Porites cylindrica*	83
Vibrio rotiferianus	Yellow disease	Yellow band disease^*d*^	*Orbicella faveolata*	81
Vibrio tubiashii	White disease^*a*^	White syndrome^*c*^	*Acropora muricata*	85
Vibrio proteolyticus	Yellow disease	Yellow band disease^*d*^	*Orbicella faveolata*	81
Unspecified *Vibrio* spp.	Black disease	Black band disease^*c*^	*Favia* spp.	86
	White disease^*a*^	Stony coral tissue loss disease^*c*^	*Montastraea cavernosa, Orbicella faveolata, Diploria labyrinthiformis,* and *Dichocoenia stokesii*	87

a Updated from Kemp et al. (2018) (88).

b Described by different authors under the names white, syndrome, pox, and/or band disease. Disease signs are manifestations of coral tissue loss and/or zooxanthellae loss or bleaching.

c Associated as a part of a bacterial consortium suspected to contain non-*Vibrio* species.

d Associated as a part of a bacterial consortium suspected to contain multiple *Vibrio* spp.

Research investigating cholera transmission in humans has demonstrated that V. cholerae cells colonize the exoskeletons of copepods where their concentration can increase to an excess of 10^4^ cells copepod^−1^ (26, 34–37). Subsequent ingestion of colonized copepods can increase the probability of ingesting a potentially pathogenic dose of the bacterium facilitating the onset of disease (38, 39). Furthermore, pre-filtration of surface water sources utilized for drinking with simple fabric mesh can reduce the occurrence of V. cholerae infections due to the reduction of colonized zooplankton (38, 40). While this *Vibrio*-zooplankton transmission pathway has been well established for V. cholerae, little research has been devoted to investigating the importance of these interactions for non-cholera *Vibrio* infections.

The work presented here investigates the viability of ingestion as a portal of entry for potentially pathogenic *Vibrio* spp. in corals. To alleviate difficulties of *ex situ* coral cultivation, a model system was employed utilizing sea anemones (*Exaiptasia pallida*) and brine shrimp (*Artemia* sp.) to mimic natural coral feeding. Prior research has demonstrated the utility of sea anemones in the genus *Exaiptasia* (formally *Aiptasia*, see Grajales & Rodriguez, 2014 [41] for reclassification) as lab-friendly surrogates for coral experimentation (41–44). Structurally, *Exaiptasia* spp. resemble large non-colonial coral polyps and feed both heterotrophically on zooplankton and autotrophically though the use of their algal symbionts (zooxanthellae) (45). Using this model system, we traced the acquisition of the putative coral pathogens V. alginolyticus, *V. mediterranei*, and V. harveyi (Table 2).[Table T3]

**TABLE 2:** Experimental *Vibrio* spp. strains used for controlled feeding studies^*a*^

Species	Strain designation	Strain isolation source	Strain citation
V. alginolyticus	ATCC 17749	Spoiled horse mackerel, Japan	89
V. harveyi	ATCC 14216	Deceased luminescent amphipod, USA	90
*V. mediterranei*	ATCC 43341	Sediment, Spain	91

a All original strains tagged with GFP using the methods described in Norfolk & Lipp (2022) (70).

## RESULTS

### *Artemia* Colonization by *Vibrio*.

**FIG 1:**
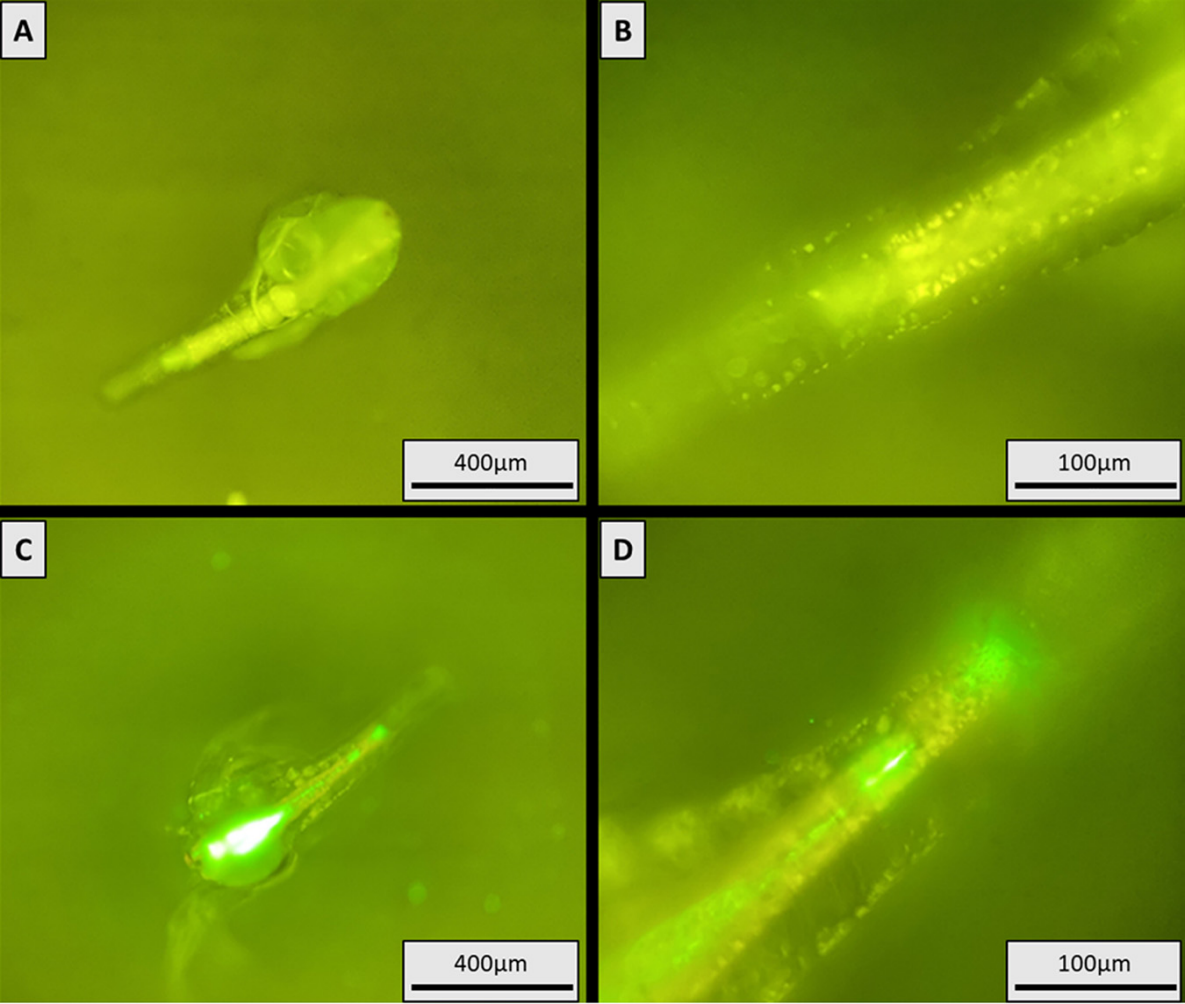
GFP V. alginolyticus colonization of *Artemia*. Cultures inoculated with ~1.1 × 10^8^ CFU. Photos taken after 18 h of exposure. (A) Unexposed *Artemia* at × 100 magnification. (B) Unexposed *Artemia* posterior at × 400 magnification. (C) Exposed *Artemia* at × 100 magnification. (D) Exposed *Artemia* posterior at × 400 magnification. Bright green fluorescence indicates GFP V. alginolyticus presence where colonization was highly concentrated throughout the length of the *Artemia* GI tract in association with ingested materials and feces. Uncolonized *Artemia* tissue appears yellow green.

Colonization experiments first assessed the ability of *Vibrio* spp. to attach to/associate with *Artemia*. Substantial colonization of *Artemia* gastrointestinal (GI) tracts was observed for all tested vibrios following overnight (18 h) exposure via ambient water at 28°C. Total colonization for each *Vibrio* spp. exposure (~250 *Artemia*) was 4.9 × 10^6^, 1.5 × 10^6^, and 7.6 × 10^6^ CFU per ~ 250 individuals for V. alginolyticus, V. harveyi, and *V. mediterranei*, respectively. These levels equate to a mean acquisition of 4.3%, 2.1%, and 50.2% of the initial exposure dose for V. alginolyticus, V. harveyi, and *V. mediterranei*, respectively (Fig. S1). It should be noted that these counts are based on culturable GFP-*Vibrio* spp. and thus may be confounded by the presence of *Vibrio* spp. in a viable but non-culturable state (VBNC) (46). However, warm water conditions and short duration exposures minimized the likelihood of VBNC formation, which is typically associated with longer duration exposure to stressful conditions (46, 47). Epifluorescence microscopy showed GFP-tagged cells were concentrated throughout the length of *Artemia* GI tracts in association with ingested material and feces (Fig. 1). GFP cells were also observed in association with *Artemia* feces following defecation. Low exoskeletal association was observed in all experimental trials, though minor attachment and/or entanglement was noted in association with *Artemia* appendages (Fig. 1). GI association was consistent across naupliiar sizes excluding the smallest, most recently hatched individuals (Fig. S2), which showed little to no GFP-*Vibrio* accumulation. Visual patterns of GI association did not differ between *Vibrio* species. No distinctive behavioral changes or swimming impairment was observed in colonized *Artemia* throughout the duration of exposure (up to 24 h).

### Uptake of *Vibrio* by *E. pallida*.

Anemone feeding studies evaluated the efficacy of an ingestion-based transmission pathway by confirming consumption of GFP-*Vibrio-*colonized *Artemia* and quantification of the acquired GFP-*Vibrio* dose. Gross observations of feeding demonstrate that *E. pallida* readily ingested *Vibrio*-colonized *Artemia*, responding rapidly with predatory tentacle behavior when *Artemia* were introduced into the microcosm water (Fig. S3). No differences in anemone feeding behavior (i.e., tentacle response) were observed for exposures using spiked and non-spiked *Artemia*.

**FIG 2:**
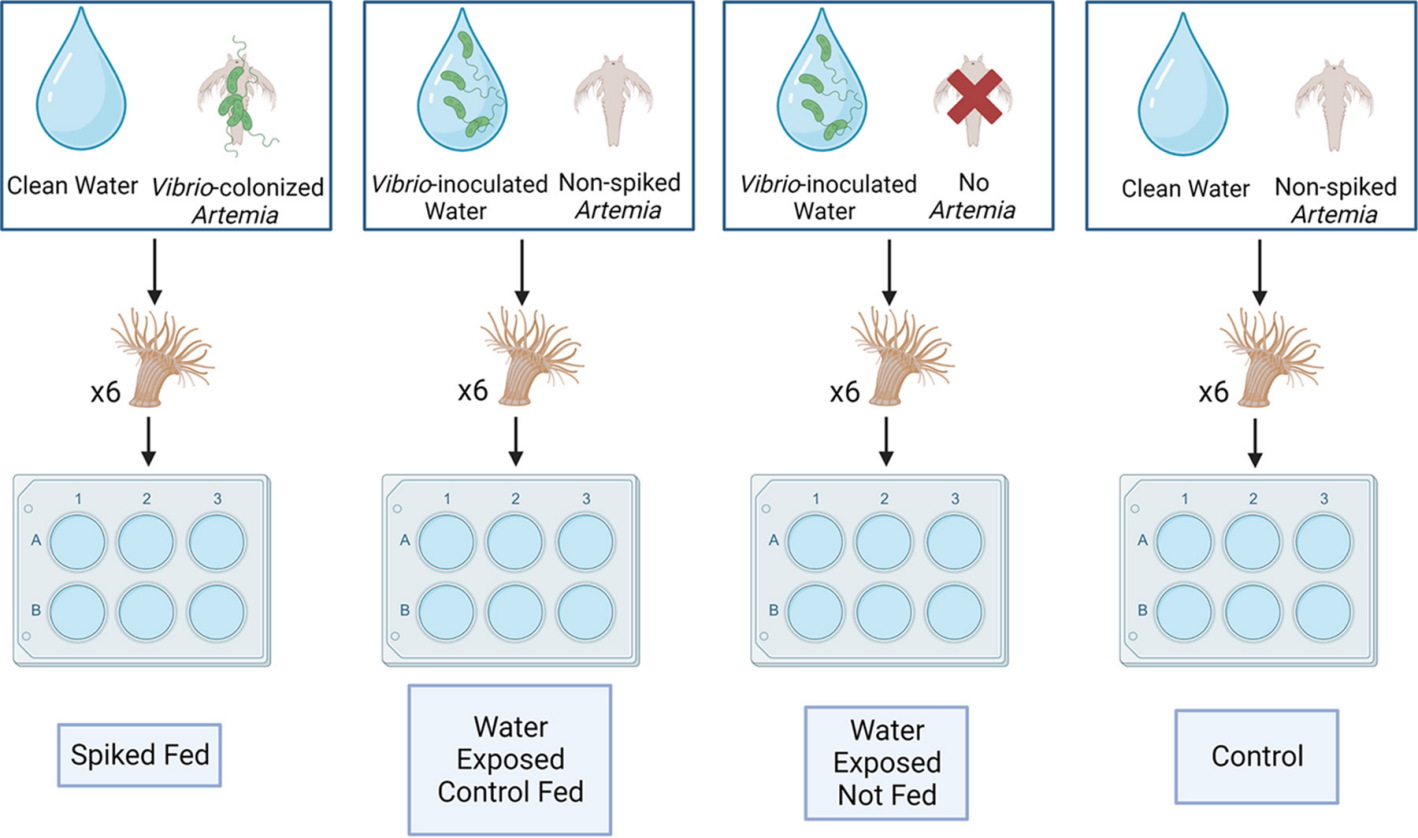
Feeding trial treatments used to expose *E. pallida* to GFP-*Vibrio*. *Artemia* administered at a concentration of ~1,000 individuals (when added). GFP-*Vibrio* administered at concentrations designated in Table 3.

**FIG 3:**
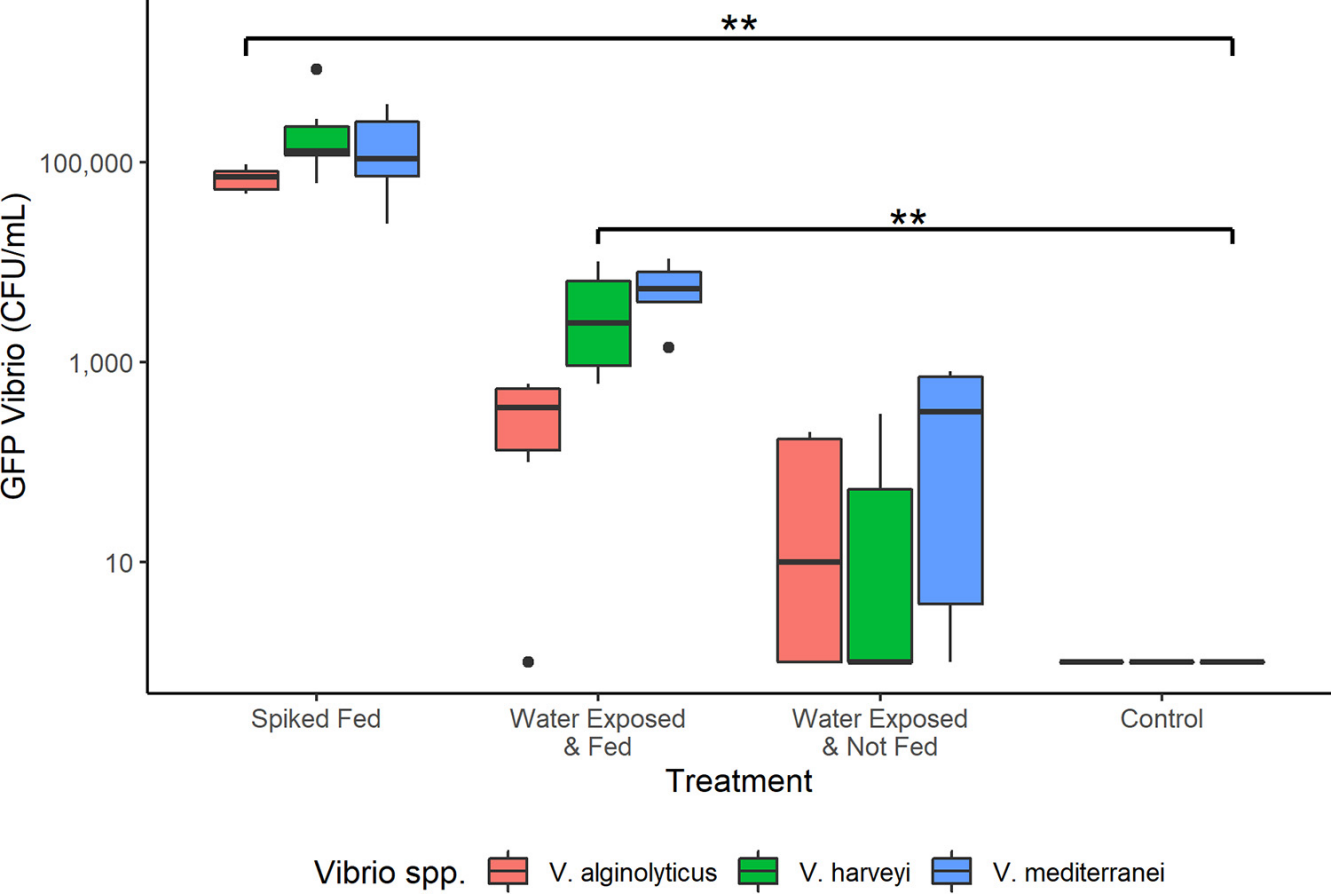
Recovered GFP-*Vibrio* spp. concentrations from anemone homogenate following completion of the controlled feeding study. Spiked fed anemones demonstrated a significantly greater GFP-*Vibrio* spp. burden compared to water exposed individuals. Spiked fed versus water exposed and fed resulted in *P*-values of 0.03, 0.013, and 0.013 and spiked fed versus water exposed not fed resulted in *P*-values of 0.028, 0.026, and 0.03 for V. alginolyticus, V. harveyi, and *V. mediterranei*, respectively. N = 6 anemones for each exposure type and *Vibrio* spp.

Assessment of the acquired dose compared 4 major feeding/exposure treatments: (i) spiked fed, where no GFP-*Vibrio* were inoculated into the microcosm water and anemones were fed with *Vibrio*-colonized *Artemia*, (ii) water exposed control fed, where GFP-*Vibrio* were inoculated into the microcosm water and anemones were fed with non-spiked *Artemia*, (iii) water exposed not fed, where GFP-*Vibrio* were inoculated into the microcosm water and no *Artemia* were added, and (iv) control, where no GFP-*Vibrio* were inoculated into the microcosm water and anemones were fed non-spiked *Artemia* (Fig. 2). Significantly greater GFP-*Vibrio* levels were observed in *E. pallida* individuals exposed via spiked *Artemia* (spiked fed) compared to individuals exposed through the ambient water, regardless of the presence of *Artemia* (i.e., all other experimental conditions). Anemone homogenate from spiked fed trials showed a mean GFP-*Vibrio* concentration of 6.9 × 10^4^, 2.6 × 10^5^, and 1.7 × 10^5^ CFU mL^−1^ for V. alginolyticus, V. harveyi, and *V. mediterranei*, respectively. Conversely, water exposed anemones showed a mean concentration of 3.3 × 10^2^ and 8.3 × 10^1^ CFU mL^−1^ for V. alginolyticus, 4.1 × 10^3^ and 8.3 × 10^1^ CFU mL^−1^ for V. harveyi, and 5.9 × 10^3^ and 3.8 × 10^2^ CFU mL^−1^ for *V. mediterranei* for water exposed control fed (non-spiked) and water exposed not fed (no *Artemia*) treatments, respectively (Table 3). These concentrations equate to a 207-fold, (*P* = 0.03), 62-fold (*P* = 0.013), and 27-fold (*P* = 0.013) increase in the GFP-*Vibrio* burden of spiked fed compared to water exposed control fed anemones and a 830-fold (*P* = 0.028), 3,108-fold (*P* = 0.026), and 435-fold (*P* = 0.030) increase in spiked fed compared to water exposed not fed anemones for V. alginolyticus, V. harveyi, and *V. mediterranei*, respectively (Fig. 3). Between the 2 water exposures, fed (non-spiked *Artemia*) anemones showed a significantly greater burden of GFP V. harveyi (*P* = 0.026) and *V. mediterranei* (*P* = 0.030) compared to non-fed anemones but did not differ significantly for V. alginolyticus (*P* = 0.51). No GFP-*Vibrio* were recovered from anemones in the control group (no exposure) or from anemone wash water (carry-over control).

**TABLE 3 T3:** GFP-*Vibrio* spp. dosing patterns, *Artemia* acquisition efficacy, *Vibrio* exposure concentration, and recovered CFU from anemone homogenate^*a*^

*Vibrio* spp.	Treatment name	Total GFP-*Vibrio* spp. Exposed to *Artemia* (CFU)^*b*^	Mean GFP-*Vibrio* spp. Carried by *Artemia* (CFU/~1000 *Artemia*)^*c*^	Total GFP-*Vibrio* spp. Inoculated into Microcosm Water (CFU)^*b*^	Mean GFP-*Vibrio* spp. Recovered from *E. pallida* Homogenate (CFU mL^−1^)
V. alginolyticus	Spiked fed	4.5 × 10^8^	2.0 × 10^7^ ± 5.6 × 10^5^	*NA*	6.9 × 10^4^ ± 7.5 × 10^3^
V. alginolyticus	Water exposed control fed	*NA*	*NA*	4.5 × 10^8^	3.3 × 10^2^ ± 1.0 × 10^2^
V. alginolyticus	Water exposed not fed	*NA*	*NA*	4.5 × 10^8^	8.3 × 10^1^ ± 4.0 × 10^1^
V. alginolyticus	Control	*NA*	*NA*	*NA*	0.0 ± 0.0
V. harveyi	Spiked fed	2.8 × 10^8^	5.9 × 10^6^ ± 2.6 × 10^5^	*NA*	2.6 × 10^5^ ± 1.1 × 10^5^
V. harveyi	Water exposed control fed	*NA*	*NA*	2.6 × 10^8^	4.1 × 10^3^ ± 1.6 × 10^3^
V. harveyi	Water exposed not fed	*NA*	*NA*	2.6 × 10^8^	8.3 × 10^1^ ± 5.4 × 10^1^
V. harveyi	Control	*NA*	*NA*	*NA*	0.0 ± 0.0
*V. mediterranei*	Spiked fed	6.1 × 10^7^	3.0 × 10^7^ ± 7.1 × 10^5^	*NA*	1.7 × 10^5^ ± 5.9 × 10^4^
*V. mediterranei*	Water exposed control fed	*NA*	*NA*	6.1 × 10^7^	5.9 × 10^3^ ± 1.4 × 10^3^
*V. mediterranei*	Water exposed not fed	*NA*	*NA*	6.1 × 10^7^	3.8 × 10^2^ ± 1.5 × 10^2^
*V. mediterranei*	Control	*NA*	*NA*	*NA*	0.0 ± 0.0

a For each experimental trial ~1000 *Artemia* (individuals) and 6 anemones (individuals) were exposed.

b Total GFP-*Vibrio* exposure represents the CFU concentration introduced to *Artemia* to promote colonization this was calculated during *Artemia* dose assessment as the sum of 4 exposure trials (~250 *Artemia* each). Initial exposures were administered at 1.1 × 10^8^, 6.9 × 10^7^, and 1.5 × 10^7^ CFU/~ 250 *Artemia* for V. alginolyticus, V. harveyi, and *V. mediterranei*, respectively. Water exposure trials were inoculated directly into the microcosm using the same concentration.

c Total GFP-*Vibrio* carried by *Artemia* represents the CFU concentration acquired by *Artemia* following exposure. This was calculated during *Artemia* dose assessment as the sum of 4 exposure trials (~250 *Artemia* each). Exposures resulted in an *Artemia*-acquired dose of 4.9 × 10^6^, 1.5 × 10^6^, and 7.6 × 10^6^ CFU/~ 250 *Artemia* for V. alginolyticus, V. harveyi, and *V. mediterranei*, respectively.

## DISCUSSION

The mucus membrane serves as the front line of defense against infection for coral species. This mucus coats the epithelia creating a semi-impermeable barrier between the coral tissue and ambient water (12, 14, 48, 49). Within this mucus layer, a variety of mutualistic and commensal microorganisms are maintained. The totality of these microbes and the coral colony are collectively known as the holobiont (50). Research has suggested that the coral-associated microbial community can confer improved fitness to the holobiont through community shifts in response to environmental change (14, 51), the production of antimicrobial compounds (52) and/or antagonistic competition with potential pathogens (50, 53, 54). Together, the physical mucus barrier combined with the biological protection of the microbial community poses a substantial challenge to the direct transmission of waterborne pathogens. To date, the majority of coral disease transmission research has focused on mechanisms of pathogen spread associated with perturbance of this mucus membrane, such as direct contact, vector-mediated (i.e., biting), and indirect transmission via preexisting lesions (11). While these studies provide important insight into the ecology of coral diseases, these transmission mechanisms are dependent on opportunistic occurrences related to host proximity and preexisting or active damage and there is substantial need to investigate transmission mechanisms related to disease emergence in uninjured corals.

Despite the presence of zooxanthellae, heterotrophic feeding is a critical component of coral nutrition, accounting for up to 35% of the daily metabolic needs of some coral species (17, 18). Corals preferentially feed on small zooplankton thus, we investigated the ability of pathogenic *Vibrio* spp. To be transmitted to a cnidarian host via ingestion following colonization of a zooplankton vector. Using sea anemones (*E. pallida*) and brine shrimp (*Artemia* spp.) to model coral feeding, we demonstrate that ingestion of *Vibrio*-spiked brine shrimp results in a significantly higher bacterial burden in recipient anemones compared to ambient water exposures, both with and without food sources (i.e., *Artemia*). These data suggest that ingestion could play a role in the transmission of certain coral pathogens. Furthermore, this mode of transmission bypasses the natural defense mechanisms of corals provided by their mucus membrane (50, 52), which may describe an important portal of entry related to pathogenic infection of uninjured corals.

Acting as our model zooplankton, *Artemia* were readily colonized by all tested *Vibrio* spp. following direct waterborne exposure, similar to previous studies in V. cholerae (34). However, the preferential colonization of the GI tract noted here differed from previously described observations where colonization was predominately observed on zooplankton exoskeletons (34–36). We hypothesize that this difference may be due to the fact that the present research was conducted *ex situ* where certain environmental determinants of zooplankton colonization (i.e., substrate limitation) may not be present and/or as impactful (21, 31, 55). While some minor exoskeletal association was observed on *Artemia* appendages, we suspect that this may be the result of incidental entanglement rather than purposeful attachment. Due to the lack of strong external association, we postulate that the colonization of *Artemia* GI tracts is the result of active ingestion of *Vibrio* spp. by nauplii occurring over prolonged interaction (≥4 h of exposure). This hypothesis is further supported by the observation that the smallest most recently hatched *Artemia* (Fig. S2) showed minimal GI colonization. At this stage of life, nauplii are nutritionally maintained through residual yolk protein and do not actively feed until they are larger (56, 57). The total *Artemia*-acquired dose remained relatively consistent for all three *Vibrio* spp. at ~ 10^6^ CFU per ~ 250 individuals. These data suggest that *Artemia* have a threshold for the maximum concentration of *Vibrio* spp. they can harbor via GI colonization.

Feeding experiments demonstrate that spiked fed anemones acquire a significantly greater GFP-*Vibrio* burden compared to water exposed individuals regardless of the presence of food. This pattern was observed across all 3 *Vibrio* spp., suggesting that ingestion of *Vibrio*-colonized zooplankton can facilitate delivery of an elevated dose of these bacteria, broadly. The higher *Vibrio* levels are likely the result of bioaccumulation of these bacteria within *Artemia* facilitating acquisition of a highly concentrated dose through targeted feeding. This is consistent with prior observations of V. cholerae carriage by copepods where ingestion of a small number of individuals may facilitate receipt of a potentially pathogenic dose (≤10^3^ cells) (34, 36). While low compared to spiked fed individuals, water exposed anemones did result in some uptake of GFP-*Vibrio* with higher levels acquired in the presence of food (non-spiked *Artemia*) than without. This observation is consistent with the findings of Gavish et al. (2021) (16) and suggests that even in the absence of *Vibrio*-colonization of food sources, active feeding and ingestion may contribute to the acquisition of *Vibrio* spp. cells from the surrounding water. It should be noted that corals are known to expel ingested pathogens as a mechanism of defense against infection (58, 59). However, during the time period of the present experiment, we did not observe any expulsion from experimental anemones.

At ambient levels, *Vibrio* spp. typically range from 10^1^ to 10^3^ CFU mL^−1^ (60) with location-specific differences in community composition driven largely by temperature and salinity (21, 25). However, *Vibrio* populations are known to be dynamic, fluctuating on a “boom-bust” cycle of growth and reduction in association with ephemeral pulses of limiting nutrients (61–63). During bloom events, total *Vibrio* can increase dramatically rising to levels 5 to 30 times greater than the typical background concentration of coastal waters (61). Prior research has shown that seasonal increases in *Vibrio* abundance facilitate increases in both free-living and zooplankton-associated abundance (64). Thus, bloom numbers could potentially promote zooplankton colonization and enhance the likelihood of transmission via ingestion during these events. While further studies on species-specific colonization rate, transmitted dose, and uptake *in situ* are needed to assess the potential importance in coral disease, we postulate that these mechanisms provide an ecological basis for foodborne transmission of certain coral pathogens.

While the scope of this research is targeted at understanding coral disease, the results of this study have broader implications for the spread of vibriosis. *Vibrio* spp. have been implicated as the causative or putative pathogens in numerous diseases of marine organisms, most notably important aquaculture species such as Pacific White Shrimp (Litopenaeus vannamei), Tiger Prawn (Penaeus monodon), Atlantic Salmon (*Salmo salar*), and Gilt-Head Sea Bream (Sparus aurata) (65–68). Zooplankton serve as the base of the marine/estuarine food web, thus there is potential for ingestion to play a role in the acquisition of these and similar pathogens. This hypothesis is supported by the work of Goulden et al. (2012) (69) who utilized a similar GFP tracking system to demonstrate that *Panulirus ornatus* (ornate spiny lobster) mortality can be facilitated by ingestion of *V. owensii*-colonized *Artemia* in aquaculture settings. Furthermore, the non-discriminant acquisition of all 3 *Vibrio* spp. in the present study suggests that this pathway may be broadly viable within the *Vibrionaceae* and warrants continued investigation of the role of ingestion in the spread of other pathogenic vibrios.

### Conclusion.

Understanding coral disease transmission is critical to the conservation of reef habitats. The present study describes a mechanistic pathway for the acquisition of coral pathogens via zooplankton ingestion using a sea anemone (*E. pallida*) and brine shrimp (*Artemia*) model system to represent coral heterotrophy. The results of this research demonstrate that ingestion of *Vibrio*-colonized *Artemia* can facilitate receipt of a significantly elevated *Vibrio* dose when compared to exposure via the water column suggesting that heterotrophy may represent an important portal of entry for certain coral pathogens. Characterization of this pathway illustrates a means by which pathogenic bacteria may bypass the natural immune defenses of corals conferred by their mucus membranes allowing for unencumbered acquisition of a pathogenic dose. This mechanism may help to explain a potential source of idiopathic infections that arise in otherwise healthy unperturbed corals.

## MATERIALS AND METHODS

### Experimental *Vibrio* strains.

Experimental *Vibrio* strains were obtained from our culture collection (E.K. Lipp, University of Georgia) and consisted of the known coral pathogens V. alginolyticus, *V. mediterranei*, and the putative coral pathogen V. harveyi (Table 2). All strains were maintained at −80°C in a 1:1 mixture of 40% glycerol (20% final concentration) and LB (Sigma-Aldrich, Miller formulation) amended to 3% wt/vol NaCl (termed LBS 3%). To revive from storage, strains were inoculated into 4 mL LBS 3% and incubated at 30°C with 100 rpm shaking agitation (New Brunswick Scientific, C24 Incubator Shaker) for 18 to 24 h.

### Brine shrimp cultures and maintenance.

*Artemia* sp. were purchased as dehydrated cysts (Premium Grade Brine Shrimp Eggs: Brine Shrimp Direct Inc., Great Salt Lake Origin). Dehydrated cysts (0.3 g) were revived in 300 mL sterile artificial seawater (35 practical salinity units [PSU] Instant Ocean, termed ASW) incubated at room temperature under mild agitation from an aquarium bubbler (Whisper 20, Aquarium Air Pump). Cysts hatching occurred within 1 to 2 days of rehydration. *Artemia* were harvested at the nauplii stage, following 1 to 2 additional days of incubation, using a sterile serological pipette. Free swimming nauplii were collected from below the water surface to reduce collection of any discarded or unhatched cysts. Any *Artemia* cultures that appeared discolored (cloudy water), produced poorly swimming nauplii, or hatched insufficiently (<75% hatching, estimated visually) were discarded.

### Anemone cultures and maintenance.

*E. pallida* anemones were purchased live (Carolina Biological Supply, #162865) and maintained in laboratory holding tanks. Holding tanks were constructed using a 6 L glass aquarium equipped with a constant-flow water filter (Aqueon QuietFlow Aquarium Power Filter 10), an in-water aquarium heater (Aqueon Pro Heater 50W), and a 445 nm aquarium light (GloFish Blue, LED Aquarium Light). Holding aquaria were maintained under the conditions outlined in Tables S2 and S3. Prior to experimentation, all anemones were transferred to holding tanks and allowed to acclimate for a minimum of 2 weeks. Anemones were monitored daily, and any deceased individuals were removed. Long-term cultures (not used for experimentation) of *E. pallida* were kept with experimental anemones to stabilize holding tank water chemistry. While in the holding tank, anemones were fed twice per week with 50 mL (~2,000 individuals) of decapsulated *Artemia*. Water changes (50% of tank volume) were peformed every 2 weeks and replaced volumetrically with fresh ASW. Intermittent tank cleaning was performed as needed using a scrub brush and/or a serological pipette to remove anemone debris and algal build-up following feeding.

### GFP tagging.

All *Vibrio* spp. used in this experiment were tagged with GFP to enable localization and quantification of the bacterium. Tagging was accomplished using the methods outlined in Norfolk & Lipp, (2022) (70). In short, a tri-parental mating assay was used to transfer a *gfp*-containing plasmid to the target *Vibrio* sp. using bacterial conjugation. In this assay, 2 strains of Escherichia coli, the helper strain carrying the conjugative plasmid pEVS104 (*tra trb* Kn^r^), and the donor strain carrying the *gfp* plasmid pVSV102 (*gfp* Kn^r^), were combined in culture with the target *Vibrio* under mild kanamycin stress to promote transfer of the *gfp* plasmid. Mating cultures were then subsequently streaked onto thiosulfate bile salts sucrose agar (TCBS) agar to remove E. coli resulting in a purified GFP-tagged *Vibrio* strain. Purification was confirmed using subsequent growth on modified mTEC agar (Difco, Fischer Scientific), an E. coli specific medium. Fluorescence of all transconjugant (GFP-tagged) *Vibrio* spp. was confirmed using fluorescence microscopy (Olympus BX41 Fluorescence Microscope). Working stocks of transconjugant strains were maintained at room temperature in deep agar stabs containing LBS 3% amended with 300 μg mL^−1^ kanamycin to ensure retention of the plasmid. GFP strains were maintained at −80°C in a 1:1 mixture of 40% glycerol and LBS 3% broth amended with 300 μg mL^−1^ kanamycin for long-term storage.

### *Artemia* colonization.

GFP-*Vibrio* spp. were revived from −80°C storage in 4 mL of LBS 3% broth amended with 300 μg mL^−1^ kanamycin and incubated at 30°C with 100 rpm of shaking agitation for 18 to 24 h. Following incubation, 1 mL of the overnight culture was pelleted by centrifugation at ~ 4,000 × *g*, the supernatant was discarded, and replaced with 1 mL of sterile 1X phosphate-buffered saline (PBS). This process was repeated three times to ensure adequate removal of residual kanamycin from the culture. Concurrently, *Artemia* cultures were grown as described above in “Brine Shrimp Cultures and Maintenance” to produce free swimming nauplii. Six mL of decapsulated nauplii (~ 250 individuals) were transferred to each well of a sterile 6-well tissue culture plate (Cellstar 6-Well Suspension Culture Plate). Each well of the culture plate was inoculated with 50 μL of washed GFP V. alginolyticus (~ 1.1 × 10^8^ CFU), V. harveyi (~ 6.9 × 10^7^ CFU), or *V. mediterranei* (~ 1.5 × 10^7^ CFU). The *Artemia*-*Vibrio* mixture was covered and incubated at 28°C under 50 rpm of shaking agitation for 18 h. This exposure duration was selected to facilitate sufficient colonization of *Artemia*, which appeared too low for experimental needs after only 3 h of exposure (Fig. S5). Following incubation, the contents of each well was collected onto a 3.0 μm polycarbonate (PCTE) membrane (Sterlitech 47 mm and 3.0 μm PCTE membranes) using vacuum filtration to capture the suspended *Artemia* while allowing any non-associated *Vibrio* cells to be discarded as flow through. The *Vibrio*-colonized *Artemia* were resuspended from the membrane by vortexing for 30 s in 6 mL of sterile ASW. Colonization or apparent attachment of GFP-labeled cells to *Artemia* nauplii was confirmed using epifluorescence microscopy. Spiked *Artemia* were then homogenized (PRO Scientific, Series 250 Homogenizer) at max speed for 120 s, and homogenate was serial diluted (10-fold in 1X PBS and spread plated using glass rattler beads [Zymo Rattler Plating Beads, 4.5 mm]) onto TCBS agar amended with 300 μg mL^−1^ kanamycin in duplicate. The addition of kanamycin to the TCBS plates selected against any non-GFP-tagged *Vibrio* cells that may have been present. TCBS plates were incubated overnight at 30°C. The resulting plate counts were used to calculate the approximate level of acquired dose.

### Uptake by *E. pallida*.

To establish a connection between ingestion and *Vibrio* uptake, a controlled feeding study was conducted to measure the level of acquired GFP-tagged *Vibrio* spp. following exposure in a microcosm. Cultures of GFP-tagged *Vibrio* spp. and *Artemia* were prepared and combined as described above in “*Artemia* Colonization” to produce spiked *Artemia*. To increase the feeding opportunity, 4 *Artemia* spike exposures (~ 250 individuals each) were combined for a total exposure of ~ 1,000 individuals resulting in a maximum feeding dose (assuming ingestion of all *Artemia*) of ~ 2.0 x10^7^ CFU, ~5.8 × 10^6^ CFU, and ~ 3.0 × 10^7^ CFU for V. alginolyticus, V. harveyi, and *V. mediterranei* trials, respectively (Table 3). Colonization of the spiked *Artemia* was confirmed prior to anemone feeding using fluorescence microscopy. Control *Artemia* (non-spiked) were prepared in tandem using the protocol but were inoculated with 50 μL of sterile 1X PBS instead of GFP-*Vibrio* spp.

Experimental microcosms were constructed to house the anemones during exposure trials. Microcosms were created using 18 × 12.5 × 5 cm Pyrex dishes filled with 750 mL of sterile ASW. Each microcosm contained a submerged six well tissue culture plate to provide substrate for *E. pallida* (N = 6 per treatment). Prior to exposure, experimental *E. pallida* were transferred to the microcosm chambers and allowed to acclimate for 18 h. Anemones used in experiments were selected based on size and consisted of individuals ranging from 1.5 cm to 3 cm (at full extension) to reduce the influence of feeding bias by large or small individuals. No discolored or wilting anemones were selected (see Fig. S4 for an example of healthy *E. pallida* appearance). Care was taken during anemone detachment to ensure no damage to the tentacles or oral disk occurred. All anemones were checked visually for viability following acclimatization and replaced as needed. Experimental exposures were administered as detailed in Table 3 for a duration of 3 h. For trials where *Artemia* were fed to *E. pallida*, anemones were observed for the first 20 min following exposure to visually confirm ingestion. Anemones were rechecked every 30 min to ensure feeding behavior was continued and to stir microcosm water (to prevent *Artemia* from congregating out of anemone reach). Following exposure, anemones were collected from the chambers, transferred into individual 50 mL conical tubes containing 40 mL of sterile ASW, and vortexed for 30 s. This process was repeated twice to remove any non-ingested GFP-*Vibrio* cells. Washed anemones were then transferred into 10 mL of sterile ASW for homogenization. A total of 100 μL of ASW was removed prior to homogenization and spread plated with glass rattler beads (Zymo Rattler Plating Beads, 4.5 mm) onto TCBS agar amended with 300 μg mL^−1^ kanamycin to ensure no ambient GFP-*Vibrio* (non-ingested) remained in the wash water (carry-over control). All anemones were then homogenized (PRO Scientific, Series 250 Homogenizer) at max speed for 120 s. *E. pallida* homogenate was serial diluted (10-fold) in 1X PBS and spread plated with glass rattler beads onto TCBS agar amended with 300 μg mL^−1^ kanamycin, in duplicate. Plates were incubated at 30°C for 18 h. The resulting plate counts (CFU/mL) were used to calculate the uptake of GFP-*Vibrio* cells by the anemones under each experimental condition. Culture results were summarized and visualized in Rstudio using the packages ‘tidyverse’ and ‘readxl.’ Feeding exposures were compared using a pairwise Wilcoxon rank-sum test with a Bonferroni correction for significance.

## References

[1] Eddy TD, Lam VW, Reygondeau G, Cisneros-Montemayor AM, Greer K, Palomares MLD, Bruno JF, Ota Y, Cheung WW. 2021. Global decline in capacity of coral reefs to provide ecosystem services. One Earth 4:1278–1285. doi:10.1016/j.oneear.2021.08.016.

[2] Wild C, Hoegh-Guldberg O, Naumann MS, Colombo-Pallotta MF, Ateweberhan M, Fitt WK, Iglesias-Prieto R, Palmer C, Bythell JC, Ortiz J-C, Loya Y, van Woesik R. 2011. Climate change impedes scleractinian corals as primary reef ecosystem engineers. Mar Freshw Res 62:205–215. doi:10.1071/MF10254.

[3] Jones GP, McCormick M, Srinivasan M, Eagle JV. 2004. Coral decline threatens fish biodiversity in marine reserves. Proc Natl Acad Sci USA 101:8251–8253. doi:10.1073/pnas.0401277101. 15150414 PMC419589

[4] Pratchett MS, Thompson CA, Hoey AS, Cowman PF, Wilson SK. 2018. Effects of coral bleaching and coral loss on the structure and function of reef fish assemblages, p. 265–293. *In* van Oppen M, Lough J. (ed). Coral bleaching. Ecological studies, vol 233. Springer, Cham.

[5] Hoegh-Guldberg O, Pendleton L, Kaup A. 2019. People and the changing nature of coral reefs. Regional Studies in Marine Science 30:100699. doi:10.1016/j.rsma.2019.100699.

[6] Harvell CD, Kim K, Burkholder JM, Colwell RR, Epstein PR, Grimes DJ, Hofmann EE, Lipp EK, Osterhaus ADME, Overstreet RM, Porter JW, Smith GW, Vasta GR. 1999. Emerging marine diseases-climate links and anthropogenic factors. Science 285:1505–1510. doi:10.1126/science.285.5433.1505. 10498537

[7] Green EP, Bruckner AW. 2000. The significance of coral disease epizootiology for coral reef conservation. Biological Conservation 96:347–361. doi:10.1016/S0006-3207(00)00073-2.

[8] Porter JW, Dustan P, Jaap WC, Patterson KL, Kosmynin V, Meier OW, Patterson ME, Parsons M, Porter JW. 2001. Patterns of spread of coral disease in the Florida Keys, p. 1–24. In(ed)The Ecology and Etiology of Newly Emerging Marine Diseases. Developments in Hydrobiology, vol 159. Springer, Dordrecht.

[9] Harvell CD, Jordán-Dahlgren E, Merkel S, Rosenberg E, Raymundo L, Smith G, Weil E, Willis B. 2007. Coral disease, environmental drivers, and the balance between coral and microbial associates. Oceanog 20:172–195. doi:10.5670/oceanog.2007.91.

[10] Montilla LM, Ascanio A, Verde A, Croquer A. 2019. Systematic review and meta-analysis of 50 years of coral disease research visualized through the scope of network theory. PeerJ 7:e7041. doi:10.7717/peerj.7041. 31198644 PMC6555395

[11] Shore A, Caldwell JM. 2019. Modes of coral disease transmission: how do diseases spread between individuals and among populations? Mar Biol 166:45. doi:10.1007/s00227-019-3490-8.

[12] Rosenberg E, Koren O, Reshef L, Efrony R, Zilber-Rosenberg I. 2007. The role of microorganisms in coral health, disease and evolution. Nat Rev Microbiol 5:355–362. doi:10.1038/nrmicro1635. 17384666

[13] Shapiro OH, Fernandez VI, Garren M, Guasto JS, Debaillon-Vesque FP, Kramarsky-Winter E, Vardi A, Stocker R. 2014. Vortical ciliary flows actively enhance mass transport in reef corals. Proc Natl Acad Sci USA 111:13391–13396. doi:10.1073/pnas.1323094111. 25192936 PMC4169935

[14] Thompson JR, Rivera HE, Closek CJ, Medina M. 2014. Microbes in the coral holobiont: partners through evolution, development, and ecological interactions. Frontiers in Cellular and Infection Microbiology 4:176. doi:10.3389/fcimb.2014.00176. 25621279 PMC4286716

[15] Certner RH, Dwyer AM, Patterson MR, Vollmer SV. 2017. Zooplankton as a potential vector for white band disease transmission in the endangered coral, *Acropora cervicornis*. PeerJ 5:e3502. doi:10.7717/peerj.3502. 28698820 PMC5502091

[16] Gavish AR, Shapiro OH, Kramarsky-Winter E, Vardi A. 2021. Microscale tracking of coral-*vibrio* interactions. ISME Communications 1:18. doi:10.1038/s43705-021-00016-0. PMC9723675 37938689

[17] Houlbrèque F, Ferrier-Pagès C. 2009. Heterotrophy in tropical scleractinian corals. Biol Rev Camb Philos Soc 84 1–17. [0.1111/j.1469-185X.2008.00058.x. doi:10.1111/j.1469-185X.2008.00058.x. 19046402

[18] Ferrier-Pagès C, Hoogenboom M, Houlbrèque F. 2010. The role of plankton in coral trophodynamics, p. 215–229. *In* Dubinsky Z, Stambler N (ed). Coral reefs: an ecosystem in transition. Springer, Dordrecht. doi:10.1007/978-94-007-0114-4.

[19] Erken M, Lutz C, McDougald D. 2015. Interactions of *Vibrio* spp. with zooplankton. Microbiol Spectr 3:VE-0003-2014. doi:10.1128/microbiolspec.VE-0003-2014. 26185068

[20] Munn CB. 2015. The role of vibrios in diseases of corals. Microbiol Spectr 3:VE-0006-2014. doi:10.1128/microbiolspec.VE-0006-2014. 26350314

[21] Takemura AF, Chien DM, Polz MF. 2014. Associations and dynamics of *Vibrionaceae* in the environment, from the genus to the population level. Front Microbiol 5:38. doi:10.3389/fmicb.2014.00038. 24575082 PMC3920100

[22] Kaneko T, Colwell RR. 1977. The annual cycle of *Vibrio parahaemolyticus* in Chesapeake bay. Microb Ecol 4:135–155. doi:10.1007/BF02014284. 24231972

[23] Heidelberg JF, Heidelberg KB, Colwell RR. 2002. Bacteria of the γ-subclass Proteobacteria associated with zooplankton in Chesapeake Bay. Appl Environ Microbiol 68:5498–5507. doi:10.1128/AEM.68.11.5498-5507.2002. 12406743 PMC129896

[24] Thompson FL, Iida T, Swings J. 2004. Biodiversity of vibrios. Microbiol Mol Biol Rev 68:403–431, table of contents. doi:10.1128/MMBR.68.3.403-431.2004. 15353563 PMC515257

[25] Turner JW, Good B, Cole D, Lipp EK. 2009. Plankton composition and environmental factors contribute to *Vibrio* seasonality. ISME J 3:1082–1092. doi:10.1038/ismej.2009.50. 19421235

[26] Magny GC, Mozumder PK, Grim CJ, Hasan NA, Naser MN, Alam M, Sack RB, Huq A, Colwell RR. 2011. Role of zooplankton diversity in *Vibrio cholerae* population dynamics and in the incidence of cholera in the Bangladesh Sundarbans. Appl Environ Microbiol 77:6125–6132. doi:10.1128/AEM.01472-10. 21764957 PMC3165371

[27] Martinez-Urtaza J, Blanco-Abad V, Rodriguez-Castro A, Ansede-Bermejo J, Miranda A, Rodriguez-Alvarez MX. 2012. Ecological determinants of the occurrence and dynamics of *Vibrio parahaemolyticus* in offshore areas. ISME J 6:994–1006. doi:10.1038/ismej.2011.156. 22094349 PMC3329108

[28] Main CR, Salvitti LR, Whereat EB, Coyne KJ. 2015. Community-level and species-specific associations between phytoplankton and particle-associated *Vibrio* species in Delaware’s inland bays. Appl Environ Microbiol 81:5703–5713. doi:10.1128/AEM.00580-15. 26070682 PMC4551232

[29] Grossart HP, Dziallas C, Leunert F, Tang KW. 2010. Bacteria dispersal by hitchhiking on zooplankton. Proc Natl Acad Sci USA 107:11959–11964. doi:10.1073/pnas.1000668107. 20547852 PMC2900670

[30] Matz C, McDougald D, Moreno AM, Yung PY, Yildiz FH, Kjelleberg S. 2005. Biofilm formation and phenotypic variation enhance predation-driven persistence of *Vibrio cholerae*. Proc Natl Acad Sci USA 102:16819–16824. doi:10.1073/pnas.0505350102. 16267135 PMC1283802

[31] Liang J, Liu J, Wang X, Lin H, Liu J, Zhou S, Sun H, Zhang XH. 2019. Spatiotemporal dynamics of free-living and particle-associated *Vibrio* communities in the Northern Chinese marginal seas. Appl Environ Microbiol 85:e00217-19. doi:10.1128/AEM.00217-19. 30824453 PMC6495765

[32] Hunt DE, Gevers D, Vahora NM, Polz MF. 2008. Conservation of the chitin utilization pathway in the *Vibrionaceae*. Appl Environ Microbiol 74:44–51. doi:10.1128/AEM.01412-07. 17933912 PMC2223224

[33] Pruzzo C, Vezzulli L, Colwell RR. 2008. Global impact of *Vibrio cholerae* interactions with chitin. Environ Microbiol 10:1400–1410. doi:10.1111/j.1462-2920.2007.01559.x. 18312392

[34] Huq A, Small EB, West PA, Huq MI, Rahman R, Colwell RR. 1983. Ecological relationships between *Vibrio cholerae* and planktonic crustacean copepods. Appl Environ Microbiol 45:275–283. doi:10.1128/aem.45.1.275-283.1983. 6337551 PMC242265

[35] Tamplin ML, Gauzens AL, Huq A, Sack DA, Colwell RR. 1990. Attachment of *Vibrio cholerae* serogroup O1 to zooplankton and phytoplankton of Bangladesh waters. Appl Environ Microbiol 56:1977–1980. doi:10.1128/aem.56.6.1977-1980.1990. 2383016 PMC184543

[36] Colwell RR. 1996. Global climate and infectious disease: the cholera paradigm. Science 274:2025–2031. doi:10.1126/science.274.5295.2025. 8953025

[37] Rawlings TK, Ruiz GM, Colwell RR. 2007. Association of *Vibrio cholerae* O1 El Tor and O139 Bengal with the copepods *Acartia tonsa* and *Eurytemora affinis*. Appl Environ Microbiol 73:7926–7933. doi:10.1128/AEM.01238-07. 17951440 PMC2168156

[38] Huq A, Xu B, Chowdhury MAR, Islam MS, Montilla R, Colwell RR. 1996. A simple filtration method to remove plankton-associated *Vibrio cholerae* in raw water supplies in developing countries. Appl Environ Microbiol 62:2508–2512. doi:10.1128/aem.62.7.2508-2512.1996. 8779590 PMC168033

[39] Nelson EJ, Harris JB, Morris JG, Jr, Calderwood SB, Camilli A. 2009. Cholera transmission: the host, pathogen and bacteriophage dynamic. Nat Rev Microbiol 7:693–702. doi:10.1038/nrmicro2204. 19756008 PMC3842031

[40] Colwell RR, Huq A, Islam MS, Aziz KMA, Yunus M, Khan NH, Mahmud A, Sack RB, Nair GB, Chakraborty J, Sack DA, Russek-Cohen E. 2003. Reduction of cholera in Bangladeshi villages by simple filtration. Proc Natl Acad Sci USA 100:1051–1055. doi:10.1073/pnas.0237386100. 12529505 PMC298724

[41] Belda-Baillie CA, Baillie BK, Maruyama T. 2002. Specificity of a model cnidarian dinoflagellate symbiosis. Biol Bull 202:74–85. doi:10.2307/1543224. 11842017

[42] Weis VM, Davy SK, Hoegh-Guldberg O, Rodriguez-Lanetty M, Pringle JR. 2008. Cell biology in model systems as the key to understanding corals. Trends Ecol Evol 23:369–376. doi:10.1016/j.tree.2008.03.004. 18501991

[43] Sunagawa S, Wilson EC, Thaler M, Smith ML, Caruso C, Pringle JR, Weis VM, Medina M, Schwarz JA. 2009. Generation and analysis of transcriptomic resources for a model system on the rise: the sea anemone *Aiptasia pallida* and its dinoflagellate endosymbiont. BMC Genomics 10:258. doi:10.1186/1471-2164-10-258. 19500365 PMC2702317

[44] Hardefeldt JM, Reichelt-Brushett AJ. 2015. Unravelling the role of zooxanthellae in the uptake and depuration of an essential metal in *Exaiptasia pallida*; an experiment using a model cnidarian. Mar Pollut Bull 96:294–303. doi:10.1016/j.marpolbul.2015.04.055. 25998725

[45] Grajales A, Rodriguez E. 2014. Morphological revision of the genus *Aiptasia* and the family *Aiptasiidae* (*Cnidaria*, *Actiniaria*, *Metridioidea*). Zootaxa 3826:55–100. doi:10.11646/zootaxa.3826.1.2. 24990039

[46] Colwell RR. 2000. Viable but nonculturable bacteria: a survival strategy. J Infect Chemother 6:121–125. doi:10.1007/pl00012151. 11810550

[47] Oliver JD, Nilsson L, Kjelleberg S. 1991. Formation of nonculturable *Vibrio vulnificus* cells and its relationship to the starvation state. Appl Environ Microbiol 57:2640–2644. doi:10.1128/aem.57.9.2640-2644.1991. 1768138 PMC183633

[48] Cooney RP, Pantos O, Le Tissier MDA, Barer MR, O'Donnell AG, Bythell JC. 2002. Characterization of the bacterial consortium associated with black band disease in coral using molecular microbiological techniques. Environ Microbiol 4:401–413. doi:10.1046/j.1462-2920.2002.00308.x. 12123476

[49] Brown BE, Bythell JC. 2005. Perspectives on mucus secretion in reef corals. Mar Ecol Prog Ser 296:291–309. doi:10.3354/meps296291.

[50] Rohwer F, Seguritan V, Azam F, Knowlton N. 2002. Diversity and distribution of coral-associated bacteria. Mar Ecol Prog Ser 243:1–10. doi:10.3354/meps243001.

[51] Reshef L, Koren O, Loya Y, Zilber-Rosenberg I, Rosenberg E. 2006. The coral probiotic hypothesis. Environ Microbiol 8:2068–2073. doi:10.1111/j.1462-2920.2006.01148.x. 17107548

[52] Shnit-Orland M, Kushmaro A. 2009. Coral mucus-associated bacteria: a possible first line of defense. FEMS Microbiol Ecol 67:371–380. doi:10.1111/j.1574-6941.2008.00644.x. 19161430

[53] Ritchie KB. 2006. Regulation of microbial populations by coral surface mucus and mucus-associated bacteria. Mar Ecol Prog Ser 322:1–14. doi:10.3354/meps322001.

[54] Teplitski M, Ritchie KB. 2009. How feasible is the biological control of coral diseases? Trends Ecol Evol 24:378–385. doi:10.1016/j.tree.2009.02.008. 19406502

[55] Worden AZ, Seidel M, Smriga S, Wick A, Malfatti F, Bartlett D, Azam F. 2006. Trophic regulation of *Vibrio cholerae* in coastal marine waters. Environ Microbiol 8:21–29. doi:10.1111/j.1462-2920.2005.00863.x. 16343318

[56] Warner AH, Beers PC, Huang FL. 1974. Biosynthesis of the diguanosine nucleotides. I. purification and properties of an enzyme from yolk platelets of brine shrimp embryos. Can J Biochem 52:231–240. doi:10.1139/o74-036. 4208243

[57] Sugumar V, Munuswamy N. 2006. Ultrastructure of cyst shell and underlying membranes of three strains of the brine shrimp *Artemia* (*Branchiopoda*: *Anostraca*) from South India. Microsc Res Tech 69:957–963. doi:10.1002/jemt.20371. 16921529

[58] Gibbin E, Gavish A, Krueger T, Kramarsky-Winter E, Shapiro O, Guiet R, Jensen L, Vardi A, Meibom A. 2019. *Vibrio coralliilyticus* infection triggers a behavioural response and perturbs nutritional exchange and tissue integrity in a symbiotic coral. ISME J 13:989–1003. doi:10.1038/s41396-018-0327-2. 30542077 PMC6462045

[59] Gao C, Garren M, Penn K, Fernandez VI, Seymour JR, Thompson JR, Raina JB, Stocker R. 2021. Coral mucus rapidly induces chemokinesis and genome-wide transcriptional shifts toward early pathogenesis in a bacterial coral pathogen. ISME J 15:3668–3682. doi:10.1038/s41396-021-01024-7. 34168314 PMC8630044

[60] Urakawa H, Rivera ING. 2006. Aquatic environment, p. 175–189. *In* Thompson FL, Austin B, Swings J (ed). The biology of vibrios. 175–189. Washington, DC., ASM Press.

[61] Westrich JR, Ebling AM, Landing WM, Joyner JL, Kemp KM, Griffin DW, Lipp EK. 2016. Saharan dust nutrients promote *Vibrio* bloom formation in marine surface waters. Proc Natl Acad Sci USA 113:5964–5969. doi:10.1073/pnas.1518080113. 27162369 PMC4889353

[62] Westrich JR, Griffin DW, Westphal DL, Lipp EK. 2018. Vibrio population dynamics in mid-Atlantic surface waters during Saharan dust events. Front Mar Sci 5. doi:10.3389/fmars.2018.00012.

[63] Borchardt T, Fisher KV, Ebling AM, Westrich JR, Xian P, Holmes CD, Landing WM, Lipp EK, Wetz MS, Ottesen EA. 2020. Saharan dust deposition initiates successional patterns among marine microbes in the Western Atlantic. Limnology & Oceanography 65:191–203. doi:10.1002/lno.11291.

[64] Carli A, Pane L, Casareto L, Bertone S, Pruzzo C. 1993. Occurrence of *Vibrio alginolyticus* in Ligurian coast rock pools (Tyrrhenian Sea, Italy) and its association with the copepod *Tigriopus fulvus* (Fisher 1860). Appl Environ Microbiol 59:1960–1962. doi:10.1128/aem.59.6.1960-1962.1993. 16348971 PMC182193

[65] Karunasagar I, Pai R, Malathi GR, Karunasagar I. 1994. Mass mortality of *Penaeus monodon* larvae due to antibiotic-resistant *Vibrio harveyi* infection. Aquaculture 128:203–209. doi:10.1016/0044-8486(94)90309-3.

[66] CMcL P, Lillehaug A. 1995. Vaccination in European salmonid aquaculture: a review of practices and prospects. Br Vet J 151:45–69. doi:10.1016/s0007-1935(05)80064-8. 7735870

[67] Balebona MC, Andreu MJ, Bordas MA, Zorrilla I, Moriñigo MA, Borrego JJ. 1998. Pathogenicity of *Vibrio alginolyticus* for cultured Gilt-Head Sea Bream (*Sparus aurata*L). Appl Environ Microbiol 64:4269–4275. doi:10.1128/AEM.64.11.4269-4275.1998. 9797276 PMC106638

[68] Zhou J, Fang W, Yang X, Zhou S, Hu L, Li X, Qi X, Su H, Xie L. 2012. A nonluminescent and highly virulent *Vibrio harveyi* strain is associated with “Bacterial White Tail Disease” of *Litopenaeus vannamei* shrimp. PLoS One 7:e29961. doi:10.1371/journal.pone.0029961. 22383954 PMC3288001

[69] Goulden EF, Hall MR, Bourne DG, Pereg LL, Høj L. 2012. Pathogenicity and infection cycle of *Vibrio owensii* in larviculture of the Ornate Spiny Lobster (*Panulirus ornatus*). Appl Environ Microbiol 78:2841–2849. doi:10.1128/AEM.07274-11. 22307306 PMC3318833

[70] Wa N, Lipp EK. 2022. Use an evaluation of a pES213-derived plasmid for the constitutive expression of gfp protein in pathogenic vibrios: a tagging tool for *in vitro* studies. Microbiol Spectr 11:e02490-22. doi:10.1128/spectrum.02490-22. 36507673 PMC9927583

[71] Ben-Haim Y, Thompson FL, Thompson CC, Cnockaert MC, Hoste B, Swings J, Rosenberg E. 2003. *Vibrio coralliilyticus* sp. nov., a temperature-dependent pathogen of the coral *Pocillopora damicornis*. Int J Syst Evol Microbiol 53:309–315. doi:10.1099/ijs.0.02402-0. 12656189

[72] Rubio-Portillo E, Yarza P, Peñalver C, Ramos-Esplá AA, Antón J. 2014. New insights into *Oculina patagonica* coral diseases and their associated *Vibrio* spp. communities. ISME J 8:1794–1807. doi:10.1038/ismej.2014.33. 24621525 PMC4139725

[73] Ushijima B, Videau P, Burger AH, Shore-Maggio A, Runyon CM, Sudek M, Aeby GS, Callahan SM. 2014. *Vibrio coralliilyticus* strain OCN008 is an etiological agent of acute *Montipora* white syndrome. Appl Environ Microbiol 80:2102–2109. doi:10.1128/AEM.03463-13. 24463971 PMC3993142

[74] Shore-Maggio A, Aeby GS, Callahan SM. 2018. Influence of salinity and sedimentation on *Vibrio* infection of the Hawaiian coral *Montipora capitata*. Dis Aquat Organ 128:63–71. doi:10.3354/dao03213. 29565254

[75] Sussman M, Willis BL, Victor S, Bourne DG. 2008. Coral pathogens identified for White Syndrome (WS) epizootics in the Indo-Pacific. PLoS One 3:e2393. doi:10.1371/journal.pone.0002393. 18560584 PMC2409975

[76] Kushmaro A, Banin E, Loya Y, Stackebrandt E, Rosenberg E. 2001. *Vibrio shiloi* sp. nov., the causative agent of bleaching of the coral *Oculina patagonica*. Int J Syst Evol Microbiol 51:1383–1388. doi:10.1099/00207713-51-4-1383. 11491336

[77] Thompson FL, Hoste B, Thompson CC, Huys G, Swings J. 2001. The coral bleaching *Vibrio shiloi* Kushmaro et al. 2001 is a later synonym of *Vibrio mediterranei* Pujalte and Garay 1986. Syst Appl Microbiol 24:516–519. doi:10.1078/0723-2020-00065. 11876359

[78] Kb R, Smith GW. 1998. Type II white-band disease. Revista de Biologica Tropical 46:199–203. https://revistas.ucr.ac.cr/index.php/rbt/article/view/29623.

[79] Gil-Agudelo DL, Smith GW, Weil E. 2006. The white band disease type II pathogen in Puerto Rico. Revista de Biología Tropical 54:59–67. http://www.scielo.sa.cr/scielo.php?script=sci_arttext&pid=S0034-77442006000600011&lng=en&nrm=iso.18457175

[80] Luna GM, Bongiorni L, Gili C, Biavasco F, Danovaro R. 2010. *Vibrio harveyi* as a causative agent of the white syndrome in tropical stony corals. Environ Microbiol Rep 2:120–127. doi:10.1111/j.1758-2229.2009.00114.x. 23766006

[81] Cervino JM, Thompson FL, Gomez-Gil B, Lorence EA, Goreau TJ, Hayes RL, Winiarski-Cervino KB, Smith GW, Hughen K, Bartels E. 2008. The *Vibrio* core group induces yellow band disease in Caribbean and Indo-Pacific reef-building corals. J Appl Microbiol 105:1658–1671. doi:10.1111/j.1365-2672.2008.03871.x. 18798767

[82] Zhenyu X, Shaowen K, Chaoqun H, Zhixiong Z, Shifeng W, Yongcan Z. 2013. First characterization of bacterial pathogen, *Vibrio alginolyticus*, for *Porites andrewsi* white syndrome in the South China Sea. PLoS One 8:e75425. doi:10.1371/journal.pone.0075425. 24086529 PMC3782433

[83] Arboleda MDM, Reichardt WT. 2010. *Vibrio* sp. causing porites ulcerative white spot disease. Dis Aquat Organ 90:93–104. doi:10.3354/dao02222. 20662365

[84] Ushijima B, Smith A, Aeby GS, Callahan SM. 2012. *Vibrio owensii* induces the tissue loss disease *Montipora* white syndrome in the Hawaiian reef coral *Montipora capitata*. PLoS One 7:e46717. doi:10.1371/journal.pone.0046717. 23056419 PMC3466290

[85] Sweet M, Bythell J. 2015. White syndrome in *Acropora muricata*: nonspecific bacterial infection and ciliate histophagy. Mol Ecol 24:1150–1159. doi:10.1111/mec.13097. 25652762 PMC4964940

[86] Arotsker L, Siboni N, Ben-Dov E, Kramarsky-Winter E, Loya Y, Kushmaro A. 2009. *Vibrio* sp. as a potentially important member of the black band disease (BBD) consortium in *Favia* sp. corals. FEMS Microbiol Ecol 70:515–524. doi:10.1111/j.1574-6941.2009.00770.x. 19780825

[87] Meyer JL, Castellanos-Gell J, Aeby GS, Häse CC, Ushijima B, Paul VJ. 2019. Microbial community shifts associated with the ongoing stony coral tissue loss disease outbreak on the Florida reef tract. Front Microbiol 10:2244. doi:10.3389/fmicb.2019.02244. 31608047 PMC6769089

[88] Kemp KM, Westrich JR, Alabady MS, Edwards ML, Lipp EK. 2018. Abundance and multilocus sequence analysis of *Vibrio* bacteria associated with diseased elkhorn coral (*Acropora palmata*) of the Florida Keys. Appl Environ Microbiol 84:e01035-17. doi:10.1128/AEM.01035-17. PMC5752851 29079623

[89] Miyamoto Y, Nakamuma K, Takizawa K. 1961. Pathogenic halophiles. Proposals of a new genus “Oceanomonas” and the amended species names. Japanese J Microbiology 5:477–486. doi:10.1111/j.1348-0421.1961.tb00225.x.

[90] Johnson FH, Shunk IV. 1936. An interesting new species of luminous bacteria. J Bacteriol 31:585–593. doi:10.1128/jb.31.6.585-593.1936. 16559916 PMC543750

[91] Pujalte MJ, Garay E. 1986. Proposal of *Vibrio mediterranei* sp. nov.: A new marine member of the genus *Vibrio*. Int J Syst Evol Microbiol 36:278–281. doi:10.1099/00207713-36-2-278.

