# Supplementary material for: Coral Disease and Ingestion: Investigating the Role of Heterotrophy in the Transmission of Pathogenic Vibrio spp. using a Sea Anemone (Exaiptasia pallida) Model System
Source: Appl Environ Microbiol. 2023 May 16;89(6):e00187-23. doi: 10.1128/aem.00187-23 (PMC10304968; doi:10.1128/aem.00187-23)
Supplement: Supplemental file 1 — Supplemental material. Download aem.00187-23-s0001.docx, DOCX file, 4.8 MB [file aem.00187-23-s0001.docx]

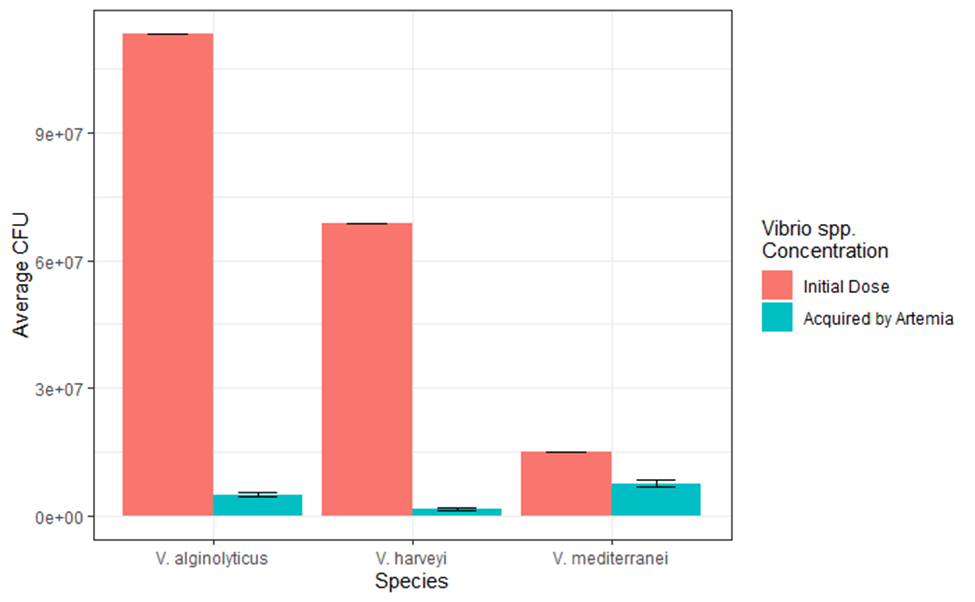


Figure S1: Average *Artemia*-acquired dose of GFP *Vibrio* spp. following an 18 h exposure via ambient water at 28^o^C under 50 rpm of shaking agitation. *V. alginolyticus* was initially dosed with 1.13 x 108 CFU resulting in an *Artemia*-acquired dose of 4.90 x 106 CFU. *V. harveyi* was initially dosed with 6.87 x 107 CFU resulting in an *Artemia*-acquired dose of 1.47 x 106 CFU*. V. mediterranei* was initially dosed with 1.51 x 107 CFU resulting in an *Artemia*-acquired dose of 7.59 x 106 CFU.


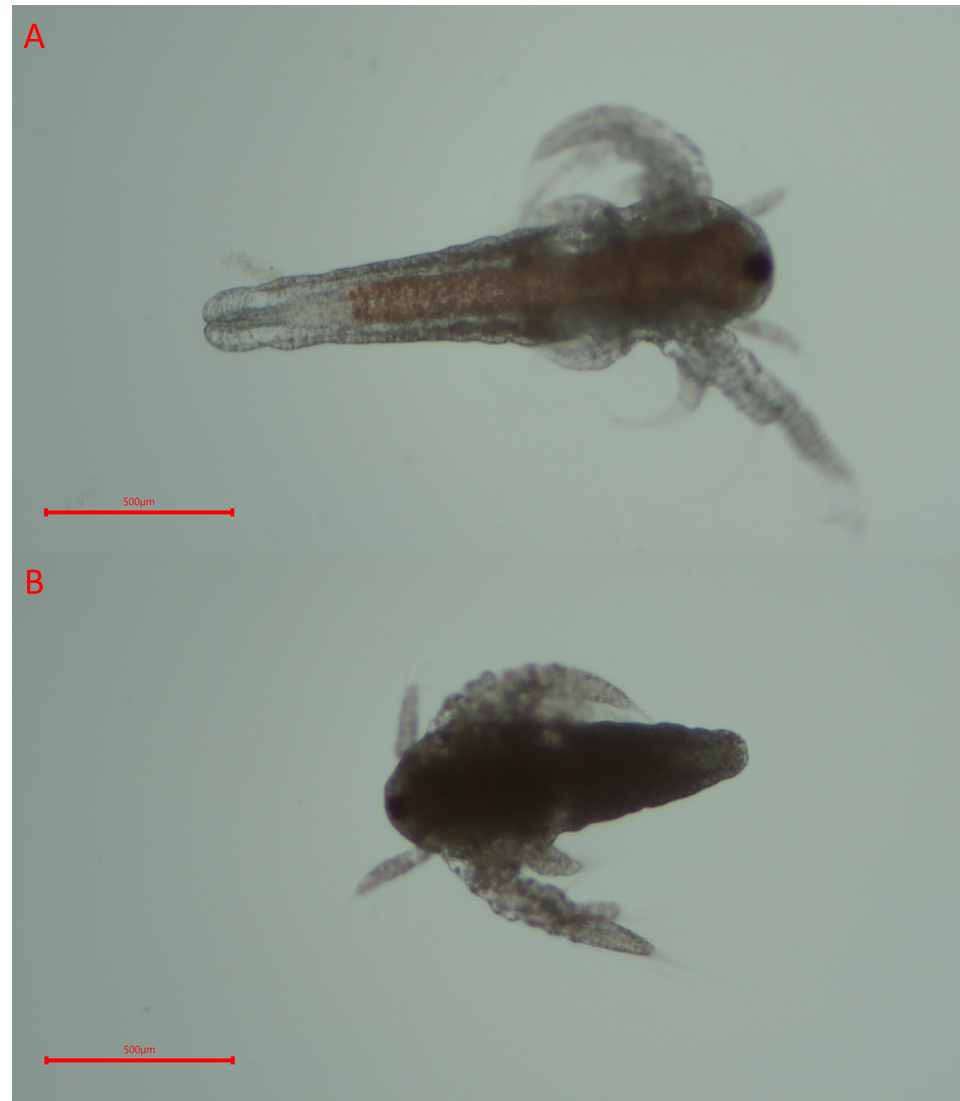


Figure S2: Hatchling verses 24 h matured *Artemia* sp. (A) 24 h matured Artemia identifiable by the longer length abdomen and more visible internal features. (B) Recently hatched (<24 h) *Artemia* sp. demonstrating shorter posterior abdominal length. Recently hatched *Artemia* sp. were not observed to accumulate a substantial burden GFP *Vibrio* spp. following exposure via water inoculation.


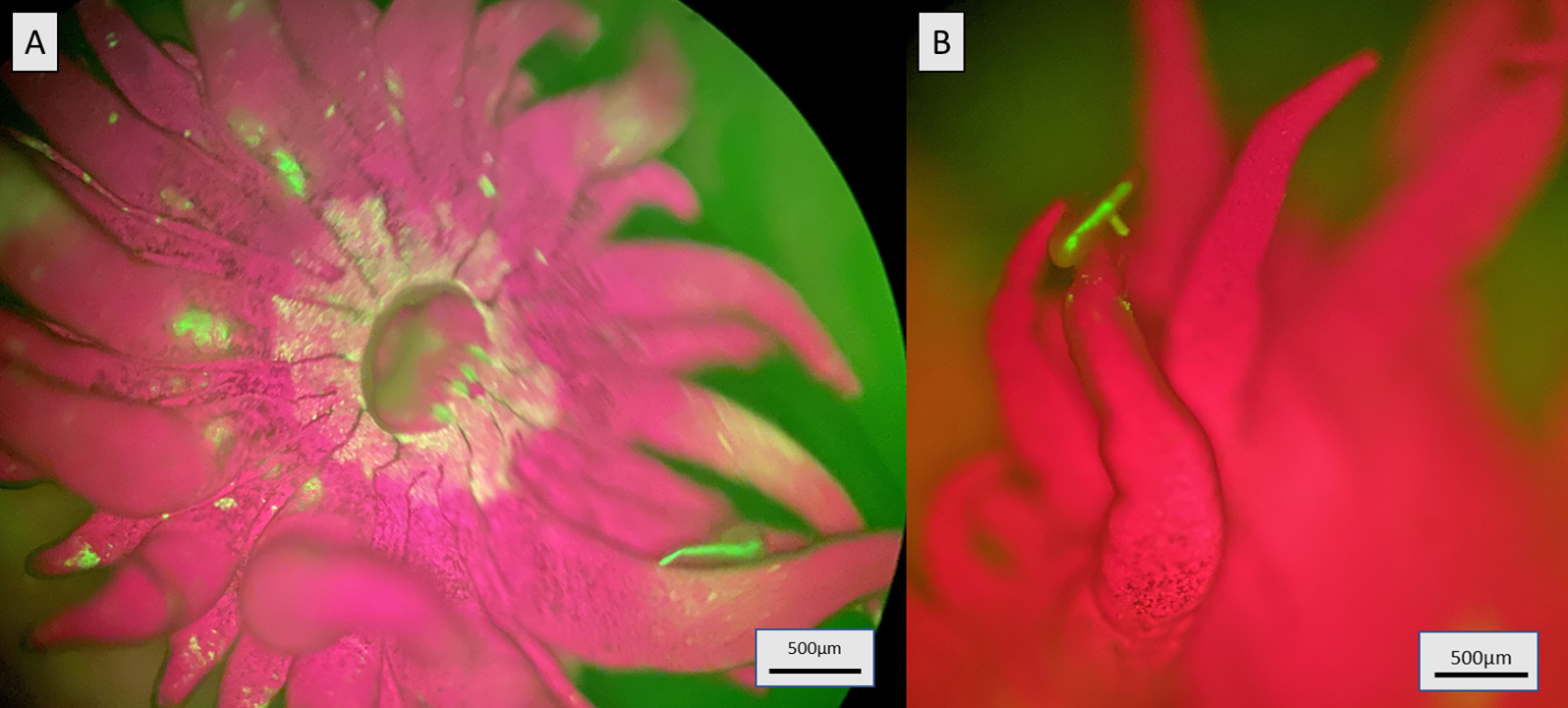


Figure S3: Images of *E. pallida* ingesting GFP-*Vibrio* (*V. alginolyticus*) spiked brine shrimp. Anemone tissue appears red/pink with intermittent green/yellow coloration around the oral disc. Artemia tissue appears translucent with bright green fluorescence concentrated throughout the length of the GI tract. (A) Image of *E. pallida* oral disc with *Artemia* captured by the lower right tentacle. (B) *E. pallida* tentacles capturing *Artemia.* Images taken at 40X magnification with 495nm excitation wavelength.

**
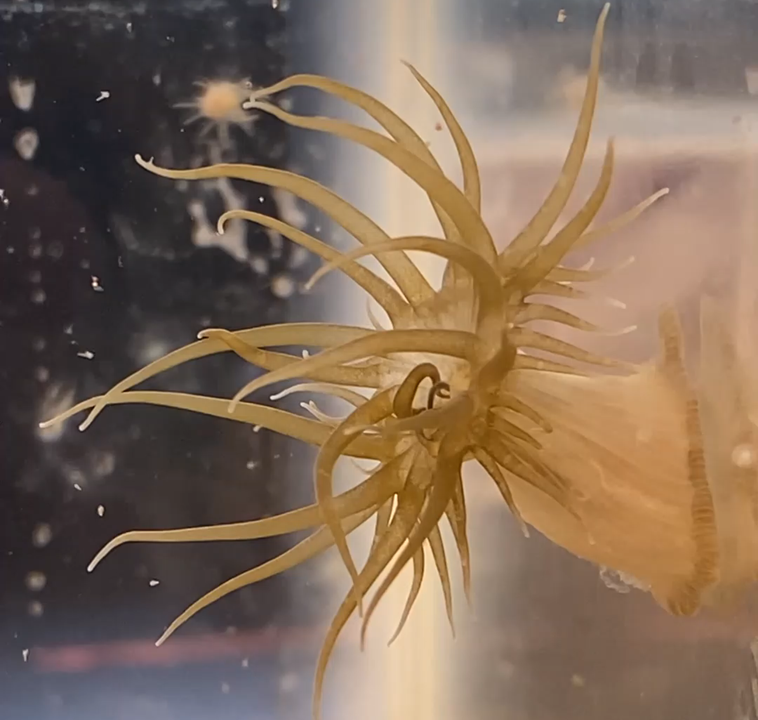
**

Figure S4: Example of a healthy *E. pallida* suitable for ingestion experimentation. Healthy anemones appear tan-brown in color with occasional dark brown striping (particularly on the tentacles). Specific shades of anemone color may vary between individuals but, should remain largely uniform. Atypical darkening or multifocal discoloration is a sign of stress. During the daytime, healthy anemones will typically rest with their tentacles outstretched flowing with the movement of the water. Intermittent curling or retraction of the tentacles is normal however, persistence in a retracted state is a sign of stress. If disturbed, healthy anemones will react to touch or sudden movement in the water in a defensive fashion by quickly retracting away from the disturbance.

Table S1: Experimental Vibrio strains used for controlled feeding studies.

| **Species** | **Strain Designation** | **Strain Isolation Source** | **Strain Citation** |
| --- | --- | --- | --- |
| *V. alginolyticus* | ATCC 17749 | Spoiled horse mackerel, Japan | Miyamoto et al., 1961 |
| *V. harveyi* | ATCC 14216 | Deceased luminescent amphipod, USA | Johnson and Shunk, 1936 |
| *V. mediterranei* | ATCC 43341 | Sediment, Spain | Pujalte & Garay, 1986 |

Table S2: Water conditions for the maintenance of *E. pallida* long-term holding tanks. Water level based on acceptable fill level 6L glass aquarium.

| **Tank Parameter** | **Acceptable Range** | **Frequency Checked** |
| --- | --- | --- |
| Temperature | 26-28^o^C | Weekly |
| Salinity | 30-35 | Weekly |
| pH | 8.0-8.5 | Weekly |
| Light Level | 15 LUX | Daily |
| Water Level | 5 ± 0.2L | Daily |

Table S3: Maintenance requirements for the management *E. pallida* holding tanks.

| **Maintenance Requirement** | **Frequency Required** | **Maintenance Details** |
| --- | --- | --- |
| Anemone Feeding | Weekly^a^ | Feeding of resident anemones with non-inoculated Artemia (50mL of decapsulated Artemia per tank) |
| Water Change | Every 2 weeks^b^ | Removal and replacement of ~50% of the total volume of artificial sea water |
| Glass Cleaning | Weekly^b^ | Gentle scrubbing of algal buildup on tank glass |
| Pump Cleaning | Monthly^b^ | Scrubbing and rinsing of pump internal compartments to reduce algal and salt buildup |
| Filter Replacement | Monthly | Replacement of charcoal pump filters |
| Heater Cleaning | Monthly^b^ | Scrubbing and rinsing of heater components to reduce algal and salt buildup |
| Light Cleaning | Monthly^b^ | Cleaning of tank lights to reduce salt buildup |
| Water Top-Off | Daily | Addition of fresh deionized water to maintain tank fill level and stabilize tank salinity due to evaporation |

^a^Feeding frequency can be increased if a tank is highly populated and/or to promote asexual reproduction of the resident anemones.

^b^Denotes minimum frequency but may be required more often based on feeding.


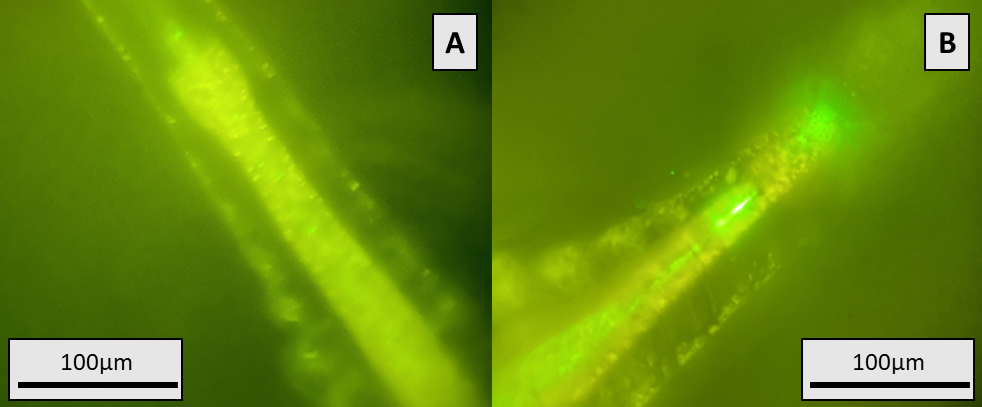


Figure S5: GFP *V. alginolyticus* colonization of *Artemia*. Cultures inoculated with ~1.1 x 108 CFU. Photos taken after 3 (A) and 18 h (B) of exposure via direct water inoculation. Exposures were maintained at 28 ^o^C with 50 rpm shaking agitation.
